# Development of an Operational Map for the 3D Printing of Phytosterol-Enriched Oleogels: Rheological Insights and Applications in Nutraceutical Design

**DOI:** 10.3390/foods14020200

**Published:** 2025-01-10

**Authors:** María Itatí De Salvo, Camila Palla, Ivana M. Cotabarren

**Affiliations:** 1 Departamento de Ingeniería Química, Universidad Nacional del Sur (UNS), Av. Alem 1253, Bahía Blanca 8000, Argentina; mdesalvo@plapiqui.edu.ar (M.I.D.S.); cpalla@plapiqui.edu.ar (C.P.); 2 Planta Piloto de Ingeniería Química-PLAPIQUI (UNS-CONICET), Camino La Carrindanga Km 7, Bahía Blanca 8000, Argentina; 3 Perfat Technologies Oy, Agnes Sjöbergin katu 2, 00790 Helsinki, Finland

**Keywords:** 3D printing, oleogel, phytosterol, rheological properties, printability, operational map

## Abstract

Three-dimensional (3D) printing attracts significant interest in the food industry for its ability to create complex structures and customize nutritional content. Printing materials, or inks, are specially formulated for food or nutraceuticals. These inks must exhibit proper rheological properties to flow smoothly during printing and form stable final structures. This study evaluates the relationship between rheological properties and printability in phytosterol-enriched monoglyceride (MG) oleogel-based inks, intended for nutraceutical applications. Key rheological factors, including gelation temperature (Tg), elastic (G′) and viscous (G″) modulus, and viscosity (µ) behavior with shear rate ($\dot{\gamma }$), were analyzed for their impact on flow behavior and post-extrusion stability. Furthermore, this study allowed the development of an operation map to predict successful printing based on material µ and Tg. Oleogels (OGs) were prepared with high-oleic sunflower oil (HOSO) and 10 wt% MG, enriched with phytosterols (PSs) at concentrations between 0 and 40 wt%. While higher PS content generally led to an increase in both Tg and µ, the 10 wt% PS mixture exhibited a different behavior, showing lower Tg and µ compared to the 0 wt% and 5 wt% PS mixtures. The optimal PS concentration was identified as 20 wt%, which exhibited optimal properties for 3D printing, with a Tg of 78.37 °C and µ values ranging from 0.013 to 0.032 Pa.s that yielded excellent flowability and adequate G′ (3.07 × 10^6^ Pa) at room temperature for self-supporting capability. These characteristics, visualized on the operational map, suggest that 20% PS OGs meet ideal criteria for successful extrusion and layered deposition in 3D printing.

## 1. Introduction

Three-dimensional food printing is a rapidly evolving technology that allows the creation of customized and intricate edible structures using specialized printers. Among its various techniques, extrusion-based 3D printing (3DP-EXT) is the most widely applied. This method involves layering edible fluids or semi-fluids (such as pastes and gels) to build specific shapes [1]. Compared to conventional food manufacturing methods, 3D food printing offers numerous advantages, including enhanced product customization, the simplification of the supply chain, and reduced material waste. Additionally, it provides the flexibility to design personalized food products. These products can be tailored to individual dietary preferences and nutritional needs [2,3,4].

In particular, 3DP-EXT by melt extrusion (ME) is an approach in which molten food materials are extruded from a moving printhead, layer-by-layer, onto a build platform. As the material is deposited, it rapidly cools and solidifies, adhering to previous layers and maintaining shape integrity [5]. Temperature control is crucial in the ME process, as the material must remain above its melting point (Tm) while inside the extruder. Upon exiting the nozzle, the material solidifies, either due to ambient cooling or an integrated cooling system, allowing for the stable stacking of layers [6]. Another 3DP-EXT method, semi-solid material extrusion (SME), involves extruding food pastes at low temperatures (such as room temperature) without melting. Suitable SME material must possess adequate mechanical strength and shear-thinning behavior to flow easily through the nozzle and retain its printed shape [6].

Lipid-based printable inks, recently explored for personalized food and nutraceutical applications, are relatively novel and offer potential for improving the nutritional and sensory attributes of 3D-printed foods [7]. To meet the specific demands of 3D printing, these inks must exhibit appropriate rheological properties for extrusion and the ability to support the weight of successive layers [8]. Rheology, the study of flow and deformation behavior of materials, is a critical factor, as it links the pre-print evaluation of ink properties to actual print performance, enhancing the predictability of print outcomes [9].

OGs, due to their specific composition and structural properties, are promising candidates for 3DP-EXT applications. These semi-solid systems, created by structuring an oil phase with gelling agents, can dissolve large amounts of lipid-soluble bioactive compounds, making them particularly relevant for nutraceutical applications [10,11]. OGs can be printed either as a semi-solid at low temperatures (SME) or in molten form (ME), requiring higher temperatures for extrusion. Printing below Tm is advantageous as it supports the stability of the printed structure, minimizes thermal degradation, and simplifies the printing setup. However, high viscosity can make processing harder, so nozzle design and extrusion pressures need to be optimized. On the other hand, printing at higher temperatures allows faster extrusion and enhanced layer adhesion but requires precise temperature control throughout the process to optimize initial and final material properties [12].

To date, only a limited number of studies have explored OG-based inks. Most studies have focused on how the type and concentration of gelators, as well as the type of oil used, influence the properties of the final product. These modifications significantly impact the physical properties of the material, influencing both its extrudability and the quality of the final printed structure. Rheology has been instrumental in assessing the behavior of these inks before the printing process, providing insights into their flow properties and stability. For instance, Shi et al. [13,14] developed inks containing potato starch, whey protein, and sunflower oil–beeswax OGs for SME. They found that G′ and G″ moduli, viscosity, and gelation temperature were sensitive to beeswax concentration, affecting both printability and structural fidelity. Similarly, Kavimughil and coworkers created ethyl cellulose-based medium-chain triglyceride OGs [11] and gelatin–gellan gum OGs enriched with bioactive compounds like curcumin and resveratrol [15] for both ME and SME processes. They observed that increasing concentrations of gellan gum and ethyl cellulose led to higher G′, G″, and complex viscosity, enhancing the inks’ extrusion properties and stability. Liu et al. [16] and Miao et al. [10] printed by SME starch-based OG capillary suspensions prepared from pea or corn starch, soybean oil, and water, analyzing G′, G″, µ, and three-interval strain structure recovery. They reported that increasing starch and water content improved the rheological properties, which was attributed to the formation of a stronger starch granule network in the oil phase held together by capillary forces. In addition, they presented high printing accuracy and the ability to maintain their shape after printing.

In our prior work [4,17], we developed high oleic sunflower oil OGs structured with MG and PS for ME applications. We characterized these inks in terms of Tg, G′, G″, apparent yield stress, and loss tangent (tan δ), and found that formulation components significantly influenced their rheological properties, impacting both the extrusion process and the quality of printed structures. This foundational work on ME with OGs has paved the way for deeper investigations into the relationship between formulation, rheology, and printability in 3D food printing applications.

Despite the progress made in the relation between material formulation and rheology, predicting the printability of food-grade materials based on their rheological characteristics remains a significant challenge [18]. High-resolution printing requires understanding the interplay between ink formulation, rheology, and printability, which is not yet fully understood for many materials, including OGs. Furthermore, empirical approaches used to optimize formulations are often labor-intensive and specific to each ink, limiting the versatility of 3D food printing [18]. Consequently, optimal print outcomes depend not only on material properties but also on the precise adjustment of printer settings. Key temperatures, such as Tm and Tg, are critical for determining nozzle temperature (T_0_) and build platform temperature (Tp), thereby enabling effective material deposition and layer stacking [2,19].

Experimental printability maps have proven valuable for exploring the interconnections between rheology, printing parameters, and printing outcomes. By varying material and process parameters, these maps delineate successful printing zones, often identifying regions where clogging, spreading, or a lack of layer support may occur. However, the extensive experimental work required for these maps limits their application for rapid ink screening. They do, however, provide valuable insights into the design principles of printable inks [20].

Also, Kim et al. [21] introduced a printability classification system, assessing the printability of common foods like yogurt, cheese, mashed potatoes, and cookie dough by evaluating their shear modulus and extrudability. Martinez Monzó et al. [22] developed predictive quadratic equations for the percentage of solid figure execution, extrusion force, and apparent viscosity in a potato puree SME process, linking these responses to formulation and printing temperature. Machine learning has also shown promise in this field. Lu et al. [18] created a machine learning model to predict the filament width, roughness, and overall printability of polysaccharide-based food inks in SME, based on formulation and rheological characteristics. More recently, Kong et al. [23] applied principal component analysis (PCA), a statistical technique that reduces data dimensions while retaining the most significant information, to classify inks formulated with tapioca starch and yeast protein based on their rheological and textural properties. This method effectively optimized their performance for SME printing. Regarding printability maps, Alvarez Castillo et al. [24] used experimental data to construct maps for plasma protein-based doughs in SME, establishing ranges of G′-frequency and viscosity-shear properties for consistent extrusion and shape retention. Oliveira et al. [25] did not present an original printability map but instead referenced the one proposed by Alvarez Castillo et al. [24] for evaluating the printability of snacks made with alga-based doughs. The printability map classified doughs based on G′, G″, and µ, assigning printability levels coded by colors. Cui et al. [26,27] designed ternary phase diagrams illustrating optimal printing conditions for gels with varying starch, thickening agent, and water contents. In a recent review, Outrequin et al. [19] synthesized data from multiple studies to generate printability figures, evaluating extrudability and shape-holding capability as a function of yield stress, G′, and tan δ for various biopolymer-based inks.

As mentioned, the link between OGs rheological properties and 3D printing behavior remains underexplored. To ensure that OGs meet the demands of 3D printing, it is essential to examine their rheological profiles in relation to extrusion performance and shape retention. Our previous studies investigated mechanical and physical properties of different formulations of MG-based OGs enriched with PS (varying the concentration of both MGs and PSs), in addition to the effect of different parameters (flow, extrusion speed, Tp, etc.) during nutraceutical 3D printing [4,17]. This study aims to investigate the relationship between rheology and printability for MG OGs inks with the addition of phytosterol (PS-enriched). To this end, an operational map is developed that predicts successful printing results. The map is constructed as a function of material viscosity, Tg, shear rates at the nozzle, and post-extrusion cooling dynamics using ME technology. To complete the operational map, a 3D transient model of temperature evolution in the printed solids during printing is proposed to optimize printing outcomes and advance the practical use of OGs in 3D food printing applications.

## 2. Materials and Methods

### 2.1. Materials

A food-grade mixture of MG, Myverol 18-08 NP, was used, composed of monoglycerides with a purity grade exceeding 90%, the specified Tm of which is 72.0 °C according to the supplier’s specification sheet (Kerry, Ireland). PS powder was a blend consisting of 34–50 wt% β-sitosterol, 17–30 wt% campesterol, 22–30 wt% stigmasterol, and approximately 2–7 wt% of other vegetable sterols (Advasterol 90F, Advanced Organic Materials, Buenos Aires, Argentina). Refined HOSO (Alsamar, Argentina) was purchased at a local grocery store.

### 2.2. Preparation of Oleogels

OGs were prepared by incorporating 10 wt% of MG in HOSO, following closely the procedure described in Cotabarren et al. [17]. To formulate printing materials with varying degrees of structuring, PS was added to the OGs at 0 to 40 wt% PS/OG. The process began by placing HOSO in a glass container system, equipped with a temperature-controlled water bath, set between 85 and 98 °C (according to the mixture PS content), with magnetic stirring at 350 rpm. After 15 min, the corresponding amount of MG was added, followed by the PS 10 min later. This mixture was kept under these conditions for 40 min to ensure the proper integration and structuring of the materials.

#### 2.2.1. Oleogel Sample Identification

To identify each formulation, samples were labeled by their PS content (Table 1). The prefix “M” denotes OG formulations with varying wt% of PS, while “O” represents a sample of pure HOSO with added PS.

### 2.3. Rheological Properties

The rheological properties of the OGs were evaluated using a Paar Physica MCR 301 Rheometer (Anton Paar GmbH, Graz, Austria) equipped with a temperature control unit, a 50 mm diameter parallel plate geometry, a 0.8 mm gap, and a computerized data acquisition system (Rheoplus/32 V3.40).

#### 2.3.1. Temperature Sweep

Oscillatory temperature sweep tests were performed on molten samples at a strain of 0.01% and an angular frequency of 1 rad/s. The samples were cooled from their preparation temperature to 5 °C at a rate of 2 °C/min. Tg was identified as the point where the G′ and G″ intersect, corresponding to a loss tangent of unity [17]. Preliminary amplitude sweep tests (strain: 0.001% to 100%; frequency: 10 rad/s; 20 °C) were conducted to determine the linear viscoelastic region.

#### 2.3.2. Viscosity

To determine the µ of the samples, a controlled shear rate rotational test was conducted, covering $\dot{\gamma }$ between 0.01 and 100 s^−1^ at a constant temperature. The tests were performed at temperatures ranging from 60 °C to 90 °C (for M0, M5, M10, and M20) or 100 °C (for M30 and M40)

### 2.4. Shear Rate at Printer Nozzle

The Hagen/Poiseuille formula provides an estimate of the $\dot{\gamma }$ for flow in capillaries, tubes, and pipes [28]. In the context of 3D printing, this formula (Equation (1)) can be adapted to approximate the $\dot{\gamma }$ as the ink flows through the syringe nozzle [29,30,31]. In this equation, *m_i_* is the average weight of the printed solid forms for each mixture, *r* is the radius of the printing nozzle, *t_i_* is the printing time, and *ρ* is the density of the OG mixture at 70 °C (value estimated based on HOSO). This calculation aims to correlate the ink’s rheological behavior with the conditions within the syringe nozzle during 3DP-EXT, aiding the selection of suitable inks.

(1) $$\dot{\gamma }=\frac{4m_{i}}{\pi r^{3}t_{i}\rho }$$

### 2.5. Heat Transfer Model in 3D Printing Process

Heat transfer is an important transport phenomenon in food production processes, where understanding conduction within solids and convection at interfaces is necessary. The separation of variables method, a well-known analytical approach, has been used to model and simulate heat transfer in food systems effectively.

**Figure 1:**
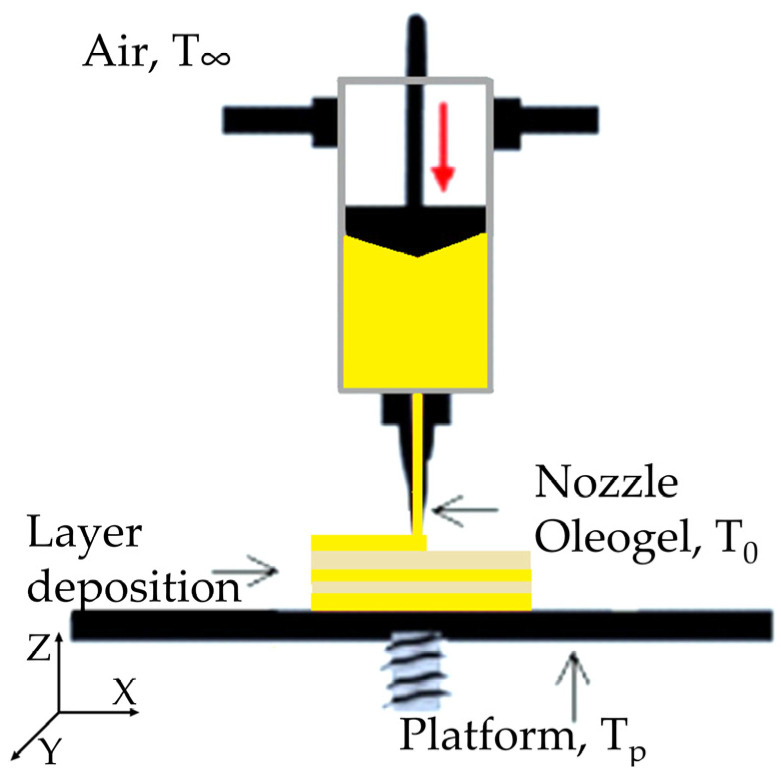
Schematic diagram of the experimental setup used in the heat transfer model, showing the boundary conditions for conduction and convection heat transfer during the cooling process of the printed material. Key components and temperature zones are labeled for clarity.

When cooling a 3D-printed solid nutraceutical, it is necessary to consider both conduction within the solid itself and convection at its interface with the surrounding air (Figure 1). In our system, the base of the solid form was in contact with a controlled Tp, while the surrounding air temperature was $T_{\infty }$. By capturing these mechanisms, the cooling process of the solid form can be more accurately modeled, providing insights into how temperature gradients develop in the structure during the printing process.

The three-dimensional heat conduction within a solid is described by the Fourier heat conduction equation (Equation (2)) [32]:

(2) $$\frac{\partial T}{\partial t}=\alpha (\frac{\partial^{2}T}{{\partial x}^{2}}+\frac{\partial^{2}T}{{\partial y}^{2}}+\frac{\partial^{2}T}{{\partial z}^{2}})$$

where T is the temperature of the solid and $\alpha =\frac{k}{\rho C_{p}}$ represents the thermal diffusivity, with k being the thermal conductivity, *ρ* the density, and Cp the specific heat capacity of the OG. Equation (2) captures how heat diffuses through the solid over time, factoring in its thermal properties.

To model heat transfer in this 3D-printed solid form, a series of boundary and initial conditions are applied. The boundary condition at the base of the solid is modeled as a fixed temperature condition, or Dirichlet condition, since the solid is in contact with a cooled platform at Tp. This results in a boundary condition expressed as Equation (3) [32]:

(3) $$T\left(x,y,z=0,t\right)=T_{p}$$

Conversely, on the side walls and the upper surface, convection occurs between the solid form and the surrounding air at $T_{\infty }$. This is modeled with a convective boundary condition, or Neumann condition, given by Equation (4) [32]:

(4) $$-k\frac{\partial T}{\partial n}=h_{\infty }(T-T_{\infty })$$

where n represents the normal direction to the surface, and $h_{\infty }$ is the convective heat transfer coefficient, assumed to be 2 W/m^2^K, a typical value for natural convection.

This condition applies to both the lateral walls and the upper surface; thus, the following expressions are used for the walls (Equation (5)) and for the upper surface at height Lz (Equation (6)), respectively:

(5) $$-k{\left.\frac{\partial T}{\partial n}\right|}_{walls}=h_{\infty }(T-T_{\infty })$$

(6) $$-k{\left.\frac{\partial T}{\partial n}\right|}_{z=Lz}=h_{\infty }(T-T_{\infty })$$

The initial condition is defined based on an initial temperature of the OG leaving the nozzle of T_0_ (Figure 1). This temperature was the average value of all measurements taken from the material exiting the nozzle in our previous 3DP-EXT experiments, as reported in De Salvo et al. [4]. This suggests that, before cooling begins, the temperature distribution is uniform throughout the sample (Equation (7)) [32]:

(7) $$T\left(x,y,z,t=0\right)=T_{0}$$

It is worth noting that, for this model, the influence of MG and PS crystallization on the energy balance is considered negligible. In fact, the phase transition heat, determined by differential scanning calorimetry in our previous work [4] represents only 6% of the total heat exchanged during the process. To solve the resulting system of partial differential equations with mixed boundary conditions, finite differences discretization was employed. This approach allowed the temporal and spatial temperature distribution within the printed nutraceutical to be determined using Python-based simulation software (Python 3.10). Heat conduction within the solid was calculated by applying a finite difference formula at each internal node, accounting for temperature values at neighboring nodes. This computation was carried out in three dimensions (x, y, z) with a time step of Δt = 0.01.

**Figure 2:**
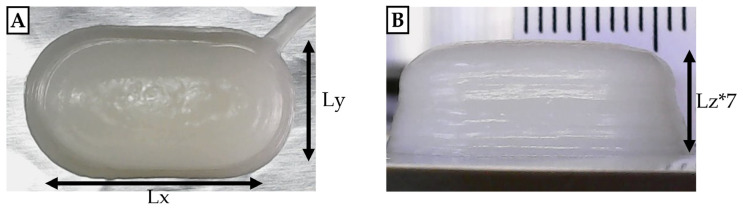
Top (**A**) and side (**B**) views of the printed solid form with dimensions for heat transfer model implementation.

The temperature at each node was iteratively updated at each time step based on changes in its nearest neighbors in the three spatial dimensions (x, y, z). For the surfaces exposed to air (top and sides), a convection formula was employed to account for heat exchange with the surrounding air. The spatial step for each mesh element was calculated as ∆i=Li(Ni−1) where L_i_ represents the length in the i-direction (Figure 2), and N_i_ represents the number of nodes in that direction (N_x_ = N_y_ = 20 and N_z_ = 10). As the solid cooled, the temperature on its exposed surface decreased, with the rate governed by the convection coefficient.

### 2.6. Three-Dimensional Printing Process

To construct the operational map and implement the heat transfer model, results from prior 3D printing experiments were utilized [4,17]. In these studies, a CAD (Computer-Aided Design) file in STL format containing the 3D digital model of the nutraceutical solid form was created using open-source software (Onshape, 2022). The design was based on the dimensions of a 1 g commercial tablet (1.874 cm in length, 0.7 cm in height, and 0.95 cm in width).

The STL file was imported into the Repetier Host V2.0.5 slicer software (Repetier, 2022), where the printing settings were adjusted accordingly. The molten mixture was loaded into the syringe, which was preheated to a temperature above Tg. Tp was set to the desired value. Once prepared, the STL file was loaded into the printer, and the extrusion process was initiated.

The printing parameters evaluated to produce the forms analyzed in this study included a flow rate of 100%; Tp = 8.5, 15, and 20 °C; T_0_ = 79.7 °C; printing speeds of 1, 3, 5, and 6 mm/s; a layer thickness of 1 mm; a nozzle diameter of 0.83 mm; and a total of seven layers.

### 2.7. Statistical Analysis

All data are presented as mean ± standard deviation. A one-way analysis of variance (ANOVA) was performed using OriginPro (2022) software to analyze the data statistically. Fisher’s test was conducted to compare the means, with a statistical significance level of *p* ≤ 0.05.

## 3. Results and Discussion

The rheological properties of inks are of critical importance in determining their feasibility for printing and obtaining a design-compliant product. In this sense, MGs, commonly used as emulsifying agents, and PSs, plant-derived compounds that promote gelation, can independently structure liquid oils. However, the combination of both gellant agents allows for the gel properties to be adjusted by changing the relative proportions of the individual components [33].

### 3.1. Rheological Analysis

**Figure 3:**
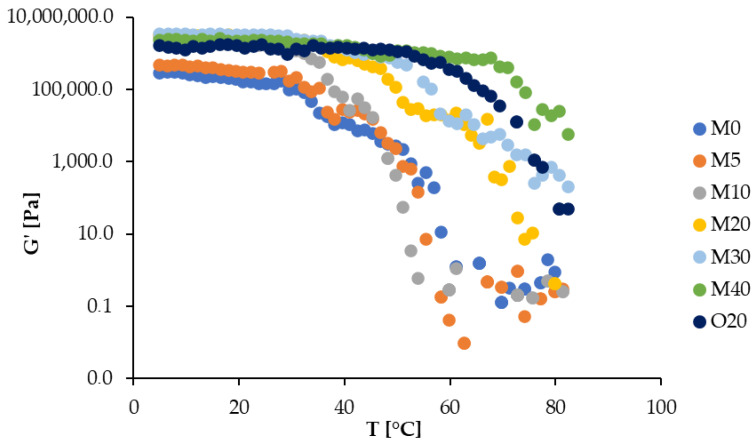
Storage modulus (G′) obtained from oscillatory temperature sweep test for various samples.

Oscillatory tests were performed to evaluate the effect of temperature on viscoelasticity. Starting from low G′ values at high temperatures, indicative of a liquid-like state, G′ increased as the temperature decreased, eventually reaching values above 1 × 10^6^ Pa (Figure 3). The G′ vs. temperature curves in Figure 3 reveal three distinct phases: (i) an initial rise in G′ at high temperatures, (ii) a slower increase above Tg, and (iii) a plateau at low temperatures. The initial G′ increase likely reflects the MG crystalline transition to a second polymorph [4,34]. The subsequent, gradual increase in G′ below Tg may be attributed to the lateral aggregation of crystalline bilayers, reinforcing the gel network [17,35]. At storage temperature (~5 °C), the G′ of multi-component OGs (except M5) greatly exceeds that of the pure MG-based OG (Figure 3), indicating stronger and more stable gels.

The tan δ ratio, defined as G″/G′, is a useful indicator of the material elasticity, classifying samples as strong gels (tan δ < 0.1), weak gels (0.1 < tan δ < 1), or viscous liquids (tan δ ≥ 1) [36]. Table 2 shows that gels M0, M5, and O2 exhibit values of 0.1 < tan δ < 1, suggesting weak gel formation. In contrast, gels M10, M20, M30, and M40 exhibit tan δ < 0.1, indicative of strong gel formation. This behavior agrees with previously reported findings for OGs structured with different vegetable oils [34,35,37]. It is important to note that tan δ was evaluated at 5 °C, acknowledging the potential changes at other temperatures, as mentioned. This value was chosen as the reference temperature due to its relevance in storage applications, ensuring a standardized comparison across samples.

**Figure 4:**
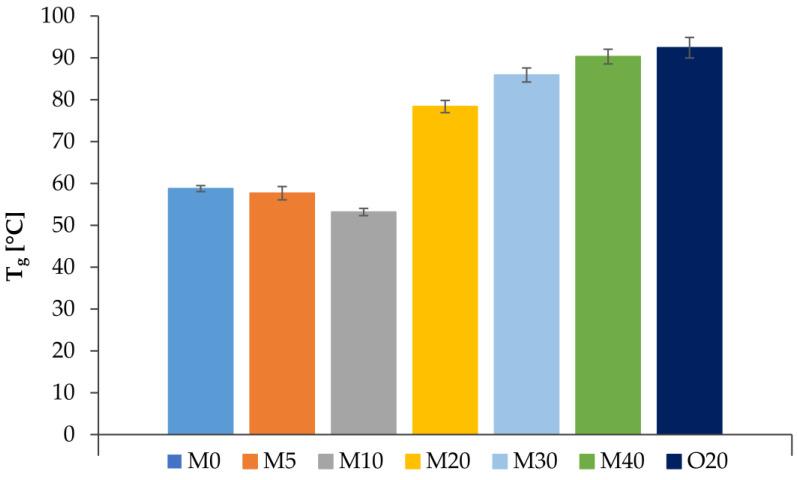
Gelation temperature (Tg) for the different mixtures.

Tg, defined as the intersection point of G′ and G″, marks the liquid-to-gel transition during the measurement process. In general, Tg increased with the addition of PS to the OGs (Figure 4), with mixtures containing PS:MG mass ratios of 2:1 or higher exhibiting higher Tg. This suggests that a threshold amount of PS is necessary to facilitate gelation, whereas lower ratios (e.g., M5 and M10) retard it, consistent with observations by Bin Sintang et al. [35]. This is because PS acts as a structuring agent that enhances molecular interactions and crystalline network formation. However, lower PS ratios likely fail to provide enough nucleation sites, leading to a weaker gel network. In fact, Bin Sintang et al. [35] observed that low-PS-ratio mixtures gelled at lower temperature than monoglycerides alone, suggesting that PSs in low concentrations inhibit the gelation process, probably due to the interruption of the ordering of monoglyceride aliphatic tails into their crystalline form.

**Figure 5:**
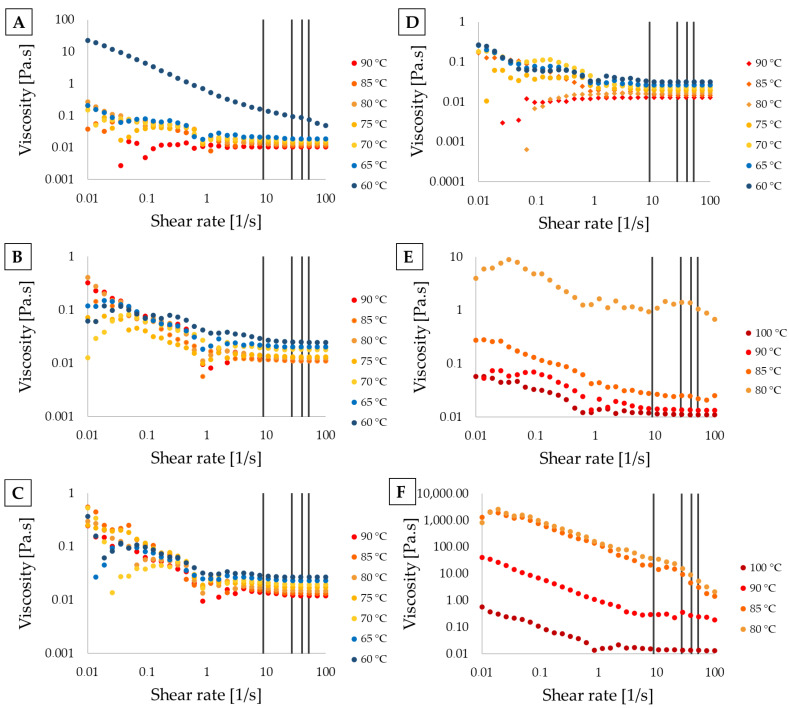
(**A**) M0; (**B**) M5; (**C**) M10; (**D**) M20; (**E**) M30; (**F**) M40. Vertical lines indicate the calculated shear velocities under specified conditions (approximately 9, 27, 40, and 52 m/s). Refer to Section 2.2.1 for coding details.

Regarding viscosity measurements, Figure 5B (M5) and Figure 5C (M10) present a typical gel behavior, characterized by a marked reduction in viscosity at high shear rates compared to low shear rates. This trend is also observed in the mono-component OG (M0, Figure 5A) at low temperatures, as well as in OGs with higher PS ratios (M30 and M40, Figure 5E,F). The reduction in viscosity correlates with gelation temperatures for gels with lower PS ratios, as depicted in Figure 4. Particularly, the M20 mixture (Figure 5D) exhibits a nearly ideal viscous flow with low and constant µ across all shear rates, suggesting that certain PS ratios promote the disintegration of crystalline groups and MG agglomerates, enhancing rheological properties compared to the mono-component OG. This finding aligns with prior work reported by [33].

The rheological response is likely due to the orientation of the suspended particles under shear forces. At rest, particles exhibiting a needle- or sheet-like geometry are randomly oriented, while shear stress induces alignment parallel to the flow, facilitating slippage and reducing resistance to movement, resulting in decreased µ [28]. Similarly, suspended agglomerates immobilize some dispersed liquid at rest, but when shear is applied, these structures disintegrate, releasing the trapped liquid and further lowering µ as the shear rate increases [28]. This effect is evident in the mixtures studied, where MG crystals, known for elongated or needle-like shapes [17,38,39], interact with PS crystals, which form fibrillar, spherulitic, or lamellar morphologies [17,33]. The resulting structure varies based on the relative proportions of each component, producing uniform crystal distribution at lower PS ratios and more pronounced aggregates at higher ratios [4,17,40].

### 3.2. Shear Rate at Printer Nozzle

The shear rate of the mixtures (M20, M30, and M40) in the 3D printing process was calculated following the methodology described in Section 2.4. Using Equation (1), we set *m_i_* = 1 g, *r* = 415 µm, and *ρ* = 0.8793 g/cm^3^, while time was varied according to the extrusion speed, as defined in previous studies [4,17]. The computed $\dot{\gamma }$ values at different extrusion speeds are summarized in Table 3.

By plotting these shear rates in Figure 5, it was confirmed that all mixtures can be feasible extruded through the nozzle due to their relatively low viscosities, enabling ink flow. However, for mixtures M30 (Figure 5E) and M40 (Figure 5F), an increase in nozzle temperature beyond 90 °C and 100 °C, respectively, would be necessary for these materials to be extruded. This temperature adjustment may exceed the operational capacity of the available commercial printers, emphasizing the need for advanced printers with higher temperature capacities to expand the range of printable formulations.

### 3.3. Heat Transfer Model in the 3D Printing Process

As discussed in Section 2.5, a numerical simulation code was developed to model the temperature evolution in the nutraceutical printed shape, represented as a three-dimensional parallelepiped. The dimensions of the solid (Figure 2) were determined based on varying printing speeds: for v = 1 mm/s, Lx = 0.01988 m, Ly = 0.01197 m, and Lz = 0.00103 m; for v = 3 mm/s, Lx = 0.01822 m, Ly = 0.01128 m, and Lz = 0.00111 m; for v = 5 mm/s, Lx = 0.01874 m, Ly = 0.0095 m, and Lz = 0.001 m; and for v = 6 mm/s, Lx = 0.01855 m, Ly = 0.01174 m, and Lz = 0.00102 m. Material properties were set as density (ρ = 880 kg/m^3^), specific heat capacity (Cp = 2400 J/kgK), and thermal conductivity (k = 0.16 W/mK) of HOSO, considering that it represents 90% of the OG formulation. The simulation time for each layer was determined by the printing speed: v_1_ = 1 mm/s, t_1_ = 314 s; v_2_ = 3 mm/s, t_2_ = 106.8 s; v_3_ = 5 mm/s, t_3_ = 72.29 s; and v_4_ = 6 mm/s, t_4_ = 55.14 s. Initial and boundary conditions were set according to Equations (3)–(7), with Tp = 8.5 °C; $T_{\infty }$ = 22.1 °C; and T_0_ = 79.7 °C.

**Figure 6:**
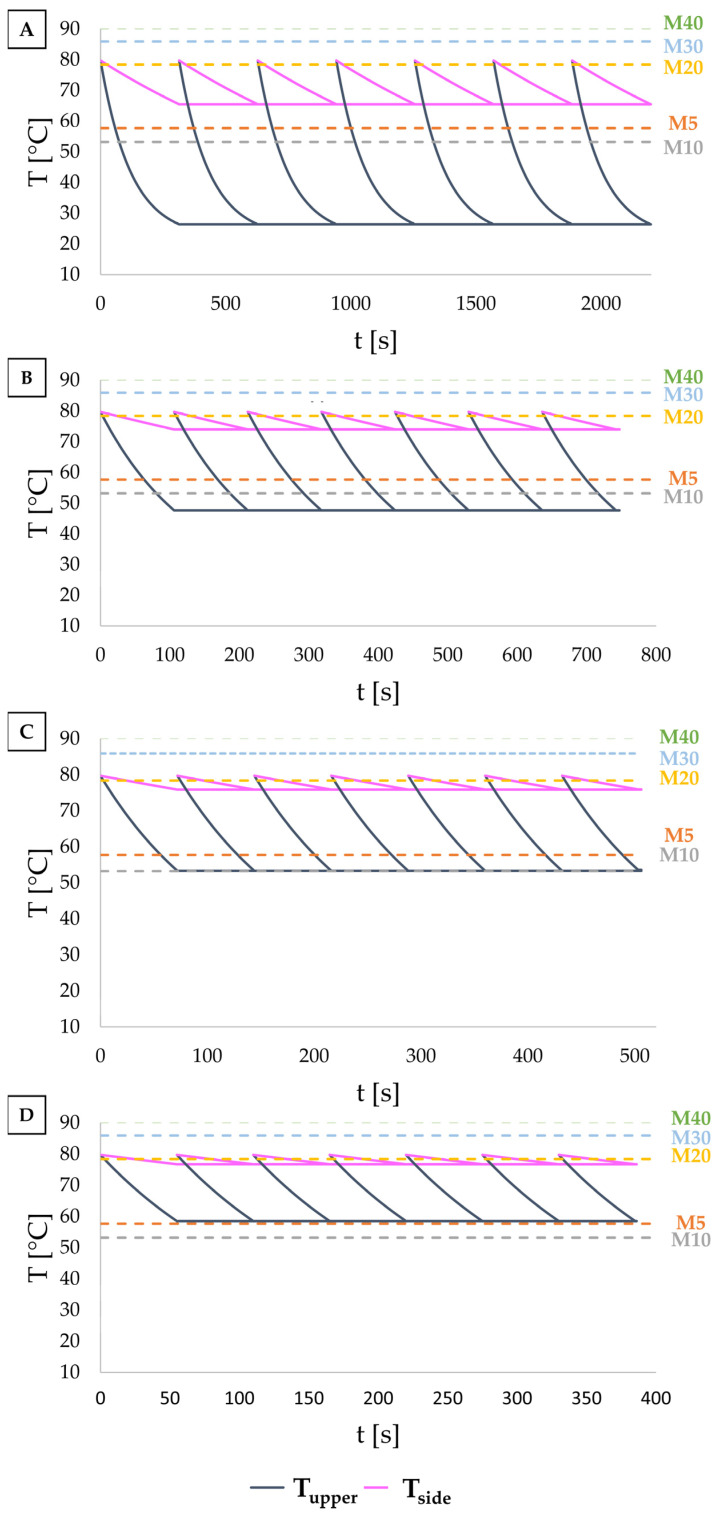
Temperature evolution over time of each printed layer at different printing speeds: (**A**) v = 1 mm/s, (**B**) v = 3 mm/s, (**C**) v = 5 mm/s, and (**D**) v = 6 mm/s. Tupper indicates the temperature at the top surface, and Tside represents the temperature at the side surfaces. Horizontal dotted lines correspond to the Tg values for the different samples. Refer to Section 2.2.1 for coding details.

To analyze temperature variation over time and across different regions of the 3D-printed nutraceutical (Figure 6), we solved the equations for heat conduction within the printed shape and for convection with the surrounding air. Starting with the first layer, the temperatures at the top surface (Tupper_j_) and side surfaces (Tside_j_) of each printed layer (j = 1 to 7) were recorded. The base temperature (Tp_j_) was updated based on the previous layer’s top surface temperature (Tupper_j−1_) after solving the heat transfer equation.

The observed thermal behavior aligns with conduction and convection mechanisms across the nutraceutical’s surfaces. Cooling is more pronounced at the base, where direct contact with the cooled plate enables higher heat transfer. This cooling effect propagates upward, with the base acting as a heat sink, drawing heat away from the interior. The top and side surfaces cool more slowly due to reliance on air convection, which is less effective than conduction. Side cooling is further limited by the smaller exposed surface area compared to the top.

In Figure 6, the temperature evolution of Tupper_j_ and Tside_j_ for each layer is shown across various speeds. The Tg values for each mixture (from Figure 4) are also indicated. As printing speed increases, the cooling time per layer decreases, resulting in final temperatures that remain closer to $T_{\infty }$. At T_0_ = 79.7 °C, mixtures M30 and M40, with Tg > T_0_, cannot be extruded as they would gelate within the syringe. In contrast, mixtures with Tg < T_0_ are suitable for printing. However, at speeds other than 1 mm/s, the Tupper values approach the Tg of M5 and M10, potentially preventing full gelation and risking structural collapse. Although T_0_ is close to Tg for M20, this mixture can still be extruded successfully [4]. Further analysis showed that Tupper_j_ < Tg for all speeds, while Tside_j_ < Tg only for v = 1 mm/s, suggesting that faster printing speeds may compromise structure stability, consistent with our previous findings [4,17].

### 3.4. Operational Map

**Figure 7:**
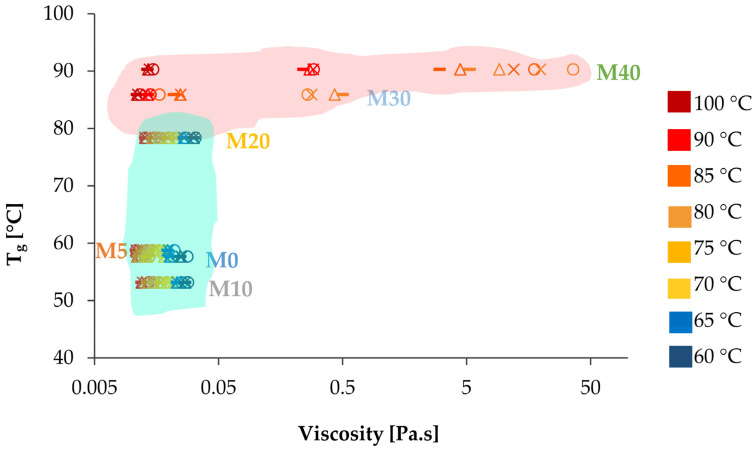
Operational map illustrating the viscosity (µ) of the mixtures at different temperatures with a constant shear rate. Symbols represent shear rates as follows: $o\;\dot{\gamma }=9\;s^{-1};\;x\;\dot{\gamma }=27\;s^{-1};\;\Delta \;\dot{\gamma }=40\;s^{-1};-\dot{\gamma }=52\;s^{-1}$. The green-shaded area indicates conditions resulting in successful prints, while the red-shaded area indicates conditions leading to unsuccessful prints. Refer to Section 2.2.1 for coding details.

An operational map was made to correlate the Tg and µ of the mixtures studied in this work with their ability to be printed. This map integrates the results from our previous studies [4,17] with the characterization of the mixtures. The operational map (Figure 7) is designed to help identify critical parameters needed for the successful extrusion-based 3D printing of structured oily inks. Results indicate that optimal print quality can be obtained with a Tg range of 50 to 80 °C and µ between 0.01 and 0.05 Pa.s, as highlighted by the green-shaded area in Figure 7. The importance of T_0_ is evident, as samples with Tg values far below T_0_ were extrudable but did not gel within the printing time (Figure 6). Therefore, achieving optimal printing conditions depends on T_0_ and the balance between the material’s fluidity and stability during deposition [4,17].

Outside the optimal range (red-shaded area in Figure 7), print quality is compromised. For example, Tg values below 50 °C lead to rapid gelation, potentially causing poor interlayer adhesion and weaker structures [41]. Conversely, Tg values above 80 °C are challenging to implement because they require elevated temperatures during printing, which can exceed the operational limits of standard 3D printers and may compromise the stability of bioactive compounds [17]. Thus, maintaining Tg within the specified range is essential for reliable extrusion.

Viscosity also plays a critical role in print quality, with values below 0.01 Pa.s resulting in uncontrolled extrusion and values above 0.05 Pa.s causing flow issues, potential blockages, and material deposition defects [17]. Ensuring that µ remains within the specified range is crucial for achieving uninterrupted, high-quality printing.

Tg and µ are associated with the formulation, as Tg and µ generally increase with higher phytosterol ratios (Figure 4 and Figure 5). Figure 7 also illustrates the impact of extrusion rate, shown as variations in shear rate. Notably, increasing the phytosterol content results in a rise in Tg and a broader range of viscosities across different shear rates and temperatures. Hence, viscosities for M30 range from 0.01 to 0.5 Pa.s, while for M40 they range from 0.01 to 36.2 Pa.s, depending on shear and temperature. In contrast, mixtures with a lower phytosterol content (e.g., M0, M5, M10, and M20–with lower Tg) exhibit a narrower viscosity range of 0.01 to 0.03 Pa.s, as shear rate and temperature vary. If the syringe heating system allows for temperatures above 80 °C, M30 and M40 could potentially be printed; however, without exceeding Tg, these mixtures could cause material blockages due to elevated µ.

In summary, this operational map represents a valuable tool for controlling and adjusting printing parameters, thereby enhancing print reproducibility and quality. Further studies should validate these results with other materials, printers, and printing conditions.

## 4. Conclusions

This study demonstrates that PS-enriched OGs exhibit rheological properties suitable for extrusion 3D printing, with the formulation containing 20% PS (M20) identified as the most suitable for this application. This formulation achieves an optimal balance between Tg and µ, allowing for controlled extrusion and stable layer deposition. The rheological behavior of M20, with an appropriate G′, ensures material flow through the nozzle while providing support for successive layers without deformation.

In contrast, formulations with higher PS concentrations (M30 and M40) showed increased Tg and µ, resulting in stronger OGs. However, this also led to challenges in extrusion through the nozzle, requiring higher extrusion temperatures. Conversely, formulations with lower PS concentrations (M5 and M10) exhibited reduced structural stability, potentially compromising the accuracy of the printed shapes.

The operational map developed in this study serves as a valuable predictive tool for correlating Tg and µ with OGs printability. Optimal operating ranges were identified as Tg between 50 and 80 °C and viscosity between 0.01 and 0.05 Pa.s, ensuring successful 3D printing. Outside the aforementioned ranges, the printing process may be adversely affected by early gelling problems or uncontrolled flows. This study establishes a framework for designing phytosterol-enriched inks with tailored rheological properties, bridging the gap between formulation science and 3D printing technology. The operational map and heat transfer insights provide valuable tools for optimizing printing parameters, enabling the production of personalized nutraceuticals and functional foods. These findings hold potential for applications in personalized nutrition and food technology, paving the way for innovations in health-oriented food products.

## Figures and Tables

**Table 1:** Nomenclature of the different oleogel formulations tested.

Mixture	MG [%]	PS [%]
M0	10	0
M5	10	5
M10	10	10
M20	10	20
M30	10	30
M40	10	40
O20	0	20

**Table 2:** Loss factor (tan δ) for different formulations.

Mixture	tan δ
**M0**	0.255
**M5**	0.343
**M10**	0.024
**M20**	0.079
**M30**	0.034
**M40**	0.055
**O20**	0.234

**Table 3:** Calculated shear rates for variable extrusion speeds.

Extrusion Speed [mm/s]	Time [s]	Shear Rate [s^−1^]
1	2198	9
3	748	27
5	506	40
6	386	52

## Data Availability

Data are contained within the article.
